# Sensory processing sensitivity predicts pilot decision time via indecisiveness: evidence from flight simulation and fNIRS

**DOI:** 10.1186/s40359-026-04402-y

**Published:** 2026-03-28

**Authors:** Kun Zhou, Yifan Zhang, Yulu Lei, Jizhang Li, Hui Tang

**Affiliations:** 1 College of Safety Science and Engineering, Civil Aviation University of China, Tianjin, 300300 People’s Republic of China; 2 School of Vocational Education, Tianjin University of Technology and Education, Tianjin, People’s Republic of China

**Keywords:** Aviation safety, Pilot decision-making, Sensory Processing Sensitivity (SPS), Indecisiveness, Dorsolateral prefrontal cortex (DLPFC), FNIRS

## Abstract

**Background:**

Timely decision-making in aviation emergencies (e.g., engine failure and runway excursion) is a critical human factor for flight safety. However, pilots vary widely in their ability to make rapid, accurate decisions under stress. Identifying individual traits and neural mechanisms underlying decision delay is essential for risk mitigation and pilot training.

**Methods:**

Fifty-one male cadet pilots were recruited and classified into high- and low-sensory processing sensitivity (SPS) groups based on their Highly Sensitive Person Scale (HSPS) scores. All participants completed simulated landing tasks under two risk conditions: a high-risk scenario (engine failure plus wind shear) and a low-risk scenario (wind shear only). The behavioural decision time, overall flight performance, and dorsolateral prefrontal cortex (DLPFC; Brodmann area 9) activation were assessed using functional near-infrared spectroscopy (fNIRS). Statistical analyses included mixed-design analyses of variance (ANOVAs), mediation analyses using the PROCESS macro, and moderated mediation models.

**Results:**

Compared with the low-SPS pilots, the high-SPS pilots had significantly longer decision times (*F* = 7.67, *p* = .008). Furthermore, a significant interaction effect between SPS and risk level was observed for flight performance; high-SPS pilots had significantly lower overall performance scores, specifically under high-risk conditions (*p* < .001). A mediation analysis indicated that indecisiveness mediated the SPS and decision time (indirect effect = 0.039, 95% CI [0.0031, 0.0792]). Moreover, BA9 activation moderated indecisiveness and decision delay (interaction *β* = 0.409, *p* = .014).

**Conclusion:**

SPS and indecisiveness jointly contribute to delayed decision-making and poor flight performance under stress, with DLPFC engagement amplifying the delay in decision-making. The findings integrate trait, behavioural, and neural factors into a unified model of aviation decision performance.

## Introduction

### Background and significance

Effective decision-making under pressure is vital to aviation safety, particularly in abnormal or emergency scenarios. Boyd and Scharf [6] analysed 1,481 fatal single-engine aircraft accidents between 1991 and 2019 and identified 846 cases associated with deficient decision-making, while the Flight Safety Foundation [16] reported that approximately 83% of runway excursion incidents could have been avoided with more timely actions. Collectively, these findings emphasise the importance of both decision accuracy and timeliness in preventing accidents.

The cockpit environment further complicates decision-making, as pilots must manage complex systems under severe time constraints while simultaneously processing continuous multisensory input from auditory warnings, visual instruments, and air traffic control communications [21, 26]. These cognitive and emotional demands are particularly pronounced during emergencies, imposing substantial strain on attentional and executive resources.

Individual differences in sensory processing sensitivity (SPS) may further exacerbate these challenges. Pilots with high SPS exhibit heightened responsiveness to sensory input, which can increase fatigue and reduce information processing efficiency [30]. Although prior research has established that excessive workload can degrade situational awareness and decision quality [2, 39], the specific role of SPS in high-stakes or contingency-related decision-making remains underexplored.

### Sensory processing sensitivity (SPS) and its impact

SPS is a personality trait characterised by heightened responsiveness to external and internal stimuli, deeper cognitive processing, greater emotional reactivity, and increased susceptibility to overstimulation [4, 20, 22, 23]. It is important to conceptually distinguish SPS from indecisiveness, as they reflect distinct stages within the cognitive process. SPS functions as a fundamental “information processing style” [4] marked by the deep and extensive processing of sensory inputs. In contrast, indecisiveness represents a specific “behavioural output” or performance deficit that emerges when this processing style conflicts with temporal constraints [17].

The mechanism that links these constructs is the cognitive cost associated with the depth of processing. High-SPS individuals automatically engage in exhaustive analysis of environmental subtleties. Under the acute time pressure characteristic of aviation emergencies, this obligatory deep processing consumes substantial cognitive resources and hinders the timely completion of the deliberation phase [1]. Consequently, the trait of high sensitivity may manifest behaviourally as indecisiveness—an inability to commit to a course of action because of information overload. Importantly, indecisiveness is increasingly conceptualised as a behavioural manifestation of difficulty in resolving internal response conflict, particularly when individuals must select between competing action alternatives under time pressure [34].

### The neural mechanism: why BA9?

Beyond behavioural mediation pathways, the neural mechanisms through which SPS influences decision timing remain largely unexplored. Empirical neuroimaging evidence has indicated that the dorsolateral prefrontal cortex (DLPFC)—particularly Brodmann area 9 (BA9)—plays a central role in uncertainty-based decision-making and higher-order cognitive control. BA9 supports goal-directed behaviour by maintaining task-relevant goals, resolving response conflict, and inhibiting prepotent responses, thereby facilitating flexible and adaptive decision-making [31, 37]. This functional profile provides a strong theoretical basis that links BA9 activity to individual variability in decisional hesitation.

Structural and functional connectivity studies further reveal a graded, topographically organised architecture within BA9. Its dorsal-rostral subregions (BA9a and BA9p) exhibit robust functional coupling with core hubs of the default mode network (DMN), including the medial prefrontal cortex, posterior cingulate cortex, and angular gyrus, which suggests a key role in integrating internally oriented, self-referential processes with externally directed, task-related cognition. Concurrently, structural connectivity between BA9 and other DLPFC subregions—as well as limbic structures such as the amygdala and hippocampus—provides an anatomical substrate for hierarchical cognitive control [8, 25]. Collectively, these network properties indicate that BA9 not only implements specific cognitive operations but also dynamically regulates the allocation of limited cognitive resources across competing information-processing streams.

Importantly, the intensity of BA9 activation may reflect the regulatory demand associated with resolving internal cognitive conflict rather than the efficiency of conflict resolution per se [14]. In highly sensitive individuals, exposure to multiple, time-pressured sensory inputs may lead to excessive BA9 recruitment as the system attempts to maintain cognitive control. Paradoxically, this heightened regulatory engagement may consume limited amounts of cognitive resources, thereby prolonging the decision-making phase. In this sense, BA9 activation both supports conflict resolution processes and functions as an index of increased demand for cognitive control, which can amplify the behavioural impact of indecisiveness on decision timing under high-load conditions.

Taken together, individual differences in BA9 activation may modulate the degree to which trait-level characteristics—such as indecisiveness—manifest as observable delays in decision-making under conditions of uncertainty or elevated risk. Within this framework, variability in BA9 activation is hypothesised to represent a neural-level mechanism through which the association among SPS, indecisiveness, and decision timing is modulated.

### Neuroimaging modality: why fNIRS?

To capture these neural dynamics in an ecologically valid setting, we employed functional near-infrared spectroscopy (fNIRS). fNIRS was chosen as the neuroimaging modality because of its unique suitability for aviation ergonomics relative to other methods. Unlike functional magnetic resonance imaging (fMRI), which imposes strict physical constraints and requires participants to remain supine, fNIRS is portable and allows data collection in realistic settings, enabling pilots to operate flight controls naturally within a cockpit [13]. Furthermore, compared with electroencephalography (EEG), fNIRS provides superior spatial resolution for mapping prefrontal cortical activity and is considerably more resilient to the electromagnetic interference and motion artefacts associated with complex manual flight tasks [11, 18, 45].

### Present study

Despite the growing interest in SPS, important literature gaps remain. Most existing studies rely primarily on self-report measures or simplified laboratory tasks, which limits ecological validity in high-stakes professional contexts. To address these limitations, the present study intends to advance the literature in three ways.

First, the study examines the influence of SPS on decision-making performance in a high-fidelity flight simulation, thereby extending SPS research to a safety–critical aviation context.

Second, by integrating fNIRS, this research provides objective neurophysiological evidence regarding DLPFC (BA9) involvement during operational decision-making. This approach allows an examination of whether heightened SPS is associated with increased prefrontal regulatory demands during conflict resolution.

Third, the study investigates indecisiveness as a specific behavioural mechanism that connects SPS to decision latency, thereby clarifying the process through which trait-level sensitivity may influence time-critical decision performance.

Guided by this framework, we propose the following hypotheses:

- H1: Higher SPS is associated with longer decision times in simulated emergency flight tasks.
- H2: Indecisiveness mediates the relationship between SPS and the decision time.
- H3: DLPFC (BA9) activation strengthens the positive association between indecisiveness and the decision time such that greater activation amplifies the impact of indecisiveness on decision delay.

## Method

### Participants

The sample size estimation using G*Power 3.1.9.7 (medium effect size, *α* = 0.05, 1–*β* = 0.80) indicated that 128 participants were required for independent-samples t tests (*d* = 0.5), 68 for simple mediation models (*f*^*2*^ = 0.15), and at least 150 for moderated mediation models [24]. A total of 132 flight cadets from the Civil Aviation University of China were initially recruited. All participants had completed both theoretical and simulator flight training, had normal vision (no history of ocular surgery), and were right-handed, as confirmed by the Edinburgh Handedness Inventory.

Based on the scores of the Highly Sensitive Person Scale, the top and bottom 27% of participants [27] were assigned to the high- and low-SPS groups, respectively. Owing to schedule constraints and training interruptions, the participants with incomplete tasks or low-quality neurobehavioural data were excluded. The final valid sample included 28 high-SPS and 23 low-SPS participants, with SPS scores differing significantly between the groups (*p* < 0.001).

Demographically, all of the participants were male flight cadets aged 21 to 23 years. To control for potential confounding variables related to flight experience, all participants were recruited from the same training cohort to ensure identical flight experience and simulator training hours according to the standardised curriculum. Furthermore, to confirm that the high- and low-SPS groups were comparable in terms of professional competency, an independent-samples t test was conducted on their flight course grades, a comprehensive measure of both academic performance and technical proficiency. No significant difference was observed between the groups (t(49) = 0.158, *p* = 0.875), indicating that the groups were well matched in terms of baseline aviation skills (see Table 1).

**Table 1:** Background Characteristics of the Final Sample by SPS Group

Characteristic	Low-SPS (n = 23)	High-SPS (n = 28)	*t*(49)	*p*
Flight Course Grade				
M (*SD*)	80.57 (5.57)	80.30 (6.09)	0.158	.875

*M* mean, *SD* standard deviation. The *p* value is two-tailed

### Apparatus and materials

#### Flight simulation platform

**Fig. 1:**
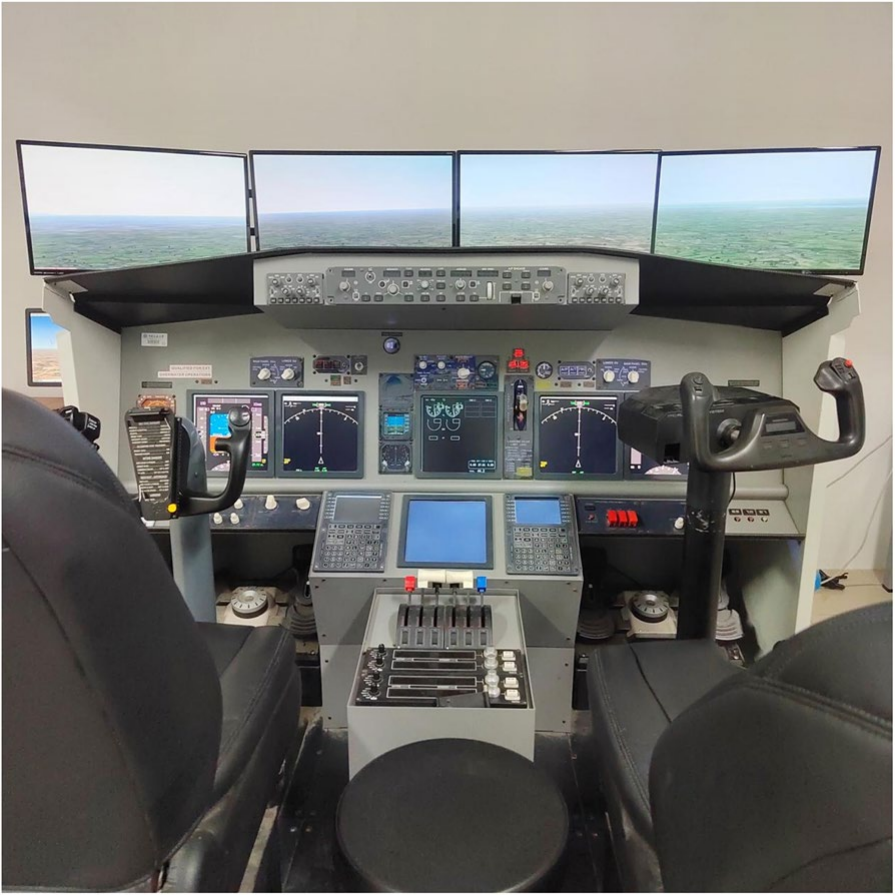
Simulated Boeing 737–800 cockpit used for the flight decision-making tasks. The setup includes a dual-seat configuration, flight controls, and a multi-screen display system

The experiment was conducted using a simulated Boeing 737–800 training cockpit (fixed-base procedure trainer). Although full-flight simulators (FFS) with motion platforms are standard for pilot handling training, a high-fidelity fixed-base simulator was deliberately chosen for this study to ensure optimal neuroimaging data quality. Motion platforms introduce substantial head movements and vibrations, which are primary sources of motion artefacts that compromise fNIRS haemodynamic signals [13, 18]. The fixed-base setup, which is equipped with a multi-screen display system and realistic flight controls (Logitech Yoke), provided sufficient visual and operational immersion to induce the necessary psychological stress (see Fig. 1).

#### Functional near-infrared spectroscopy (fNIRS)

Haemodynamic responses were recorded using a Brite MKII system (Artinis Medical Systems, Netherlands) that operated at wavelengths of 760 and 850 nm. Data were continuously acquired at a sampling rate of 25 Hz. The optode montage initially comprised 10 sources and 8 detectors, theoretically generating 24 channels that cover the prefrontal cortex. Following data quality control procedures, four channels were excluded because of consistently poor signal-to-noise ratios across the participants, which left 20 valid channels for the final statistical analysis.

**Fig. 2:**
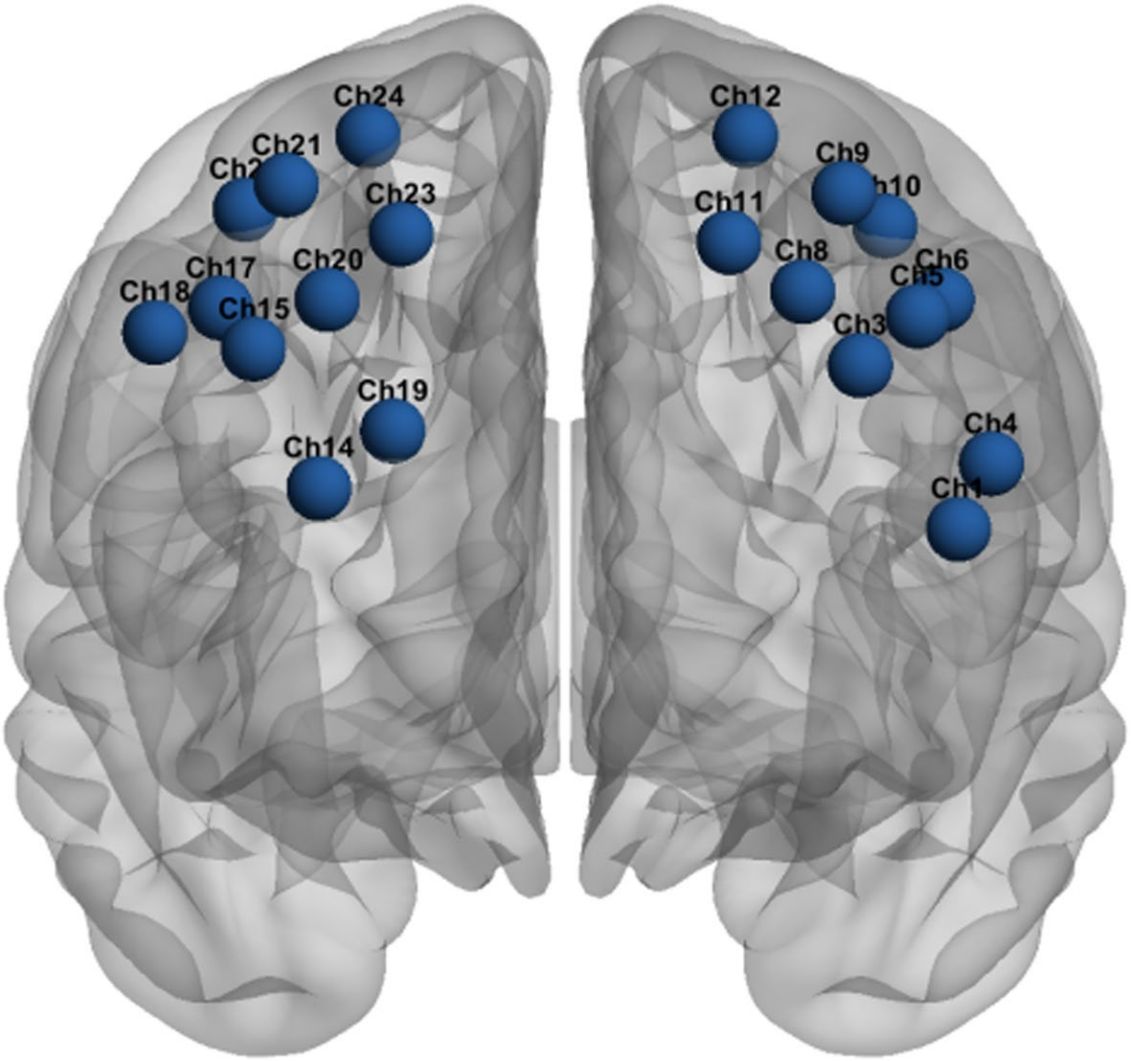
Spatial distribution of the valid fNIRS channels mapped onto a standard cortical surface. Note. The blue spheres represent the MNI coordinates of the 20 channels retained for analysis following quality control procedures. Although the original montage consisted of 24 channels, four were excluded because of signal artefacts and are therefore not depicted. The channel identifiers correspond to the anatomical definitions listed in Table 2

The spatial configuration of these 20 valid channels is shown in Fig. 2. The optode positions were registered to the Montreal Neurological Institute (MNI) standard space using head model registration, and the channels were subsequently mapped to specific Brodmann areas (BAs) using the Talairach Client plugin (University of Texas Health Science Center at San Antonio). The MNI coordinates and corresponding anatomical labels are summarised in Table 2. The brain activation for each Brodmann area was operationalised as the average activation across all valid channels located within that specific region. The differential pathlength factor (DPF) was age-calibrated for each participant.

**Table 2:** Brodmann Area Mapping of the Valid fNIRS Channels Using MNI Coordinates

Brodmann Area	Valid fNIRS Channels
AntPFC(BA10)	Ch1, Ch14
DLPFC(dorsal)(BA9)	Ch3, Ch5, Ch8, Ch15, Ch19, Ch20
DLPFC(lat)(BA46)	Ch4
Front Eye Fields(BA8)	Ch6, Ch9, Ch11, Ch17, Ch18, Ch21, Ch22, Ch23
PreMot + SuppMot(BA6)	Ch10, Ch12, Ch24

*MNI* Montreal Neurological Institute, *AntPFC* anterior prefrontal cortex, *DLPFC* dorsolateral prefrontal cortex, *FEF* frontal eye fields, *PreMot* + *SuppMot* premotor and supplementary motor cortex. “Valid channels” refers to the retained channels for analysis after quality control procedures excluded noisy channels

### Questionnaires

Three scales were employed to measure the key psychological constructs.

1) Sensory Processing Sensitivity (SPS): The Highly Sensitive Person Scale (HSPS; [4]) was used to measure SPS. The instrument consists of 27 items rated on a 7-point Likert scale (1 = not at all, 7 = extremely), with higher scores reflecting greater sensitivity. The scale has demonstrated high internal consistency (Cronbach’s *α* = 0.87) and adequate discriminant validity relative to measures of anxiety and depression [5].

2) Indecisiveness: To better capture decision-making under operational time pressure, the present study adapted the 15-item *Indecisiveness Scale* (IS; [17]). The revision aimed to enhance the contextual relevance and item clarity within aviation emergency scenarios. Based on an expert evaluation by certified flight instructors and feedback obtained during the pilot-testing phase, three items (Items 4, 7, and 13) were removed because their content reflected routine daily life decisions (e.g., clothing selection) that lacked relevance to flight operations. The removal of these items was intended to improve ecological validity and response appropriateness within high-responsibility, time-constrained decision environments.

To evaluate the psychometric properties of the adapted 12-item scale, data from the initial screening phase (*N* = 135) drawn from the same cadet cohort were used. Exploratory factor analysis (EFA) was conducted to examine construct validity. The dataset demonstrated strong suitability for a factor analysis (Kaiser–Meyer–Olkin measure = 0.84; Bartlett’s test of sphericity: *χ*^*2*^(66) = 419.07, *p* < 0.001).

The EFA yielded a four-factor solution that explained 64.3% of the total variance. All of the retained items demonstrated satisfactory primary loadings and minimal cross-loadings. The structure comprised the following factors:

- Decision-related anxiety and distraction (e.g., “I worry about making the wrong choice”; factor loadings ranged from 0.655 to 0.763);
- Confidence in decision outcomes (e.g., “I feel confident that it is a good decision”; loadings ranged from 0.602 to 0.763);
- Decision efficiency (e.g., “I make decisions quickly”; loadings ranged from 0.688 to 0.818); and.
- Delay tendency (representing the behavioural postponement of decision commitment; loading = 0.892).

This factor structure reflects distinct yet complementary components of operational decision-making, and it captures both cognitive–emotional interference and behavioural execution efficiency under emergency conditions. The adapted scale demonstrated satisfactory internal consistency within the present sample (Cronbach’s *α* = 0.77).

3) Subjective Stress Assessment (Manipulation Check). To verify the effectiveness of the risk manipulation, a single-item subjective stress rating was employed. Immediately after the completion of each flight simulation block (high- and low-risk), the participants were asked to rate their perceived stress during the preceding task on a 5-point Likert scale ranging from 1 (“No stress at all”) to 5 (“Extremely stressful”). Responses were collected using a paper-based questionnaire administered by the experimenter during the inter-trial break.

### Experimental design

This study employed a 2 (SPS group: high vs. low) × 2 (task risk level: high vs. low) mixed factorial design. Task risk level served as a within-subjects factor, whereas the SPS group functioned as a between-subjects factor. To control for potential order effects (e.g., fatigue or practice), the presentation sequence of the two risk blocks was counterbalanced across participants. Specifically, 50% of the participants completed the high-risk block first followed by the low-risk block (order AB), while the remaining 50% completed the reverse sequence (order BA). This full counterbalancing ensured that the effects of the risk condition were not confounded by the temporal sequence of the experiment.

### Experimental tasks

In this study, two flight tasks were designed to simulate decision-making under varying emergency risk levels, namely, high- and low-risk. All tasks were conducted under clear weather and optimal runway conditions, ensuring that the risk arose solely from the experimental manipulations.

**Fig. 3:**
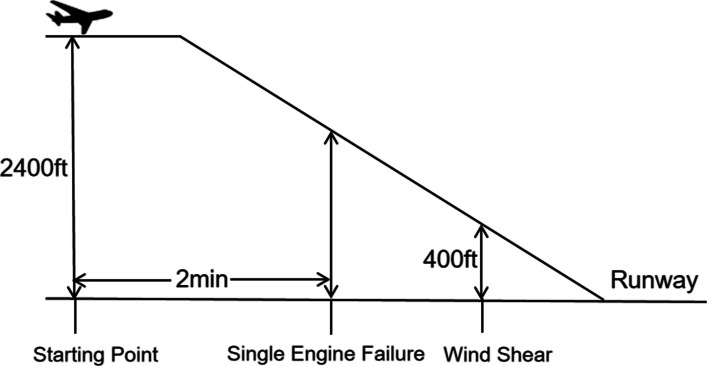
Experimental task flow for high- and low-risk landing scenarios. Note. All tasks originated at 2,400 ft AGL with a heading of 340° (initial approach fix). Key events included the following: (1) high-risk condition: left engine failure (N1 fan speed = 0, with auditory/EICAS alerts) triggered 120 s after task initiation and moderate wind shear (vertical: ± 15 ft/s; horizontal: ± 20 knots) applied for 5 s at 400 ft AGL; (2) low-risk condition: no engine failure and identical wind shear disturbance at 400 ft AGL. The cessation of wind shear marked the specific starting point for measuring the decision time

In the high-risk task, a left-engine failure occurred 120 s after task onset, accompanied by auditory warnings and Engine Indication and Crew Alerting System (EICAS) alerts. A moderate wind shear (vertical ± 15 ft/s; horizontal ± 20 knots) was applied for 5 s at 400 feet above ground level (AGL) to simulate severe meteorological interference during the final approach. In the low-risk task, no engine failure occurred, but the same wind shear at 400 feet AGL was applied to represent a more common, manageable flight challenge. The task flow and key event annotations are illustrated in Fig. 3.

### Experimental procedure

During the experiment, the participants acted as the pilot-in-command (captain) and collaborated with a male experimenter who served as a co-pilot. The flight task involved a standard approach and landing at Tianjin Binhai International Airport (RWY34L). The initial parameters were set according to the official approach chart: initial approach fix (IAF), heading 340°, altitude 2,400 ft, and a speed limit of 204 knots.

After providing written informed consent, the participants were fitted with the fNIRS cap and calibrated. Detailed instructions regarding the task objectives and operational requirements were provided. The participants were required to continuously monitor their aircraft status and perceived risk and on the basis of their assessment, decide whether to continue the approach or execute a go-around manoeuvre by verbally reporting their decision.

**Fig. 4:**
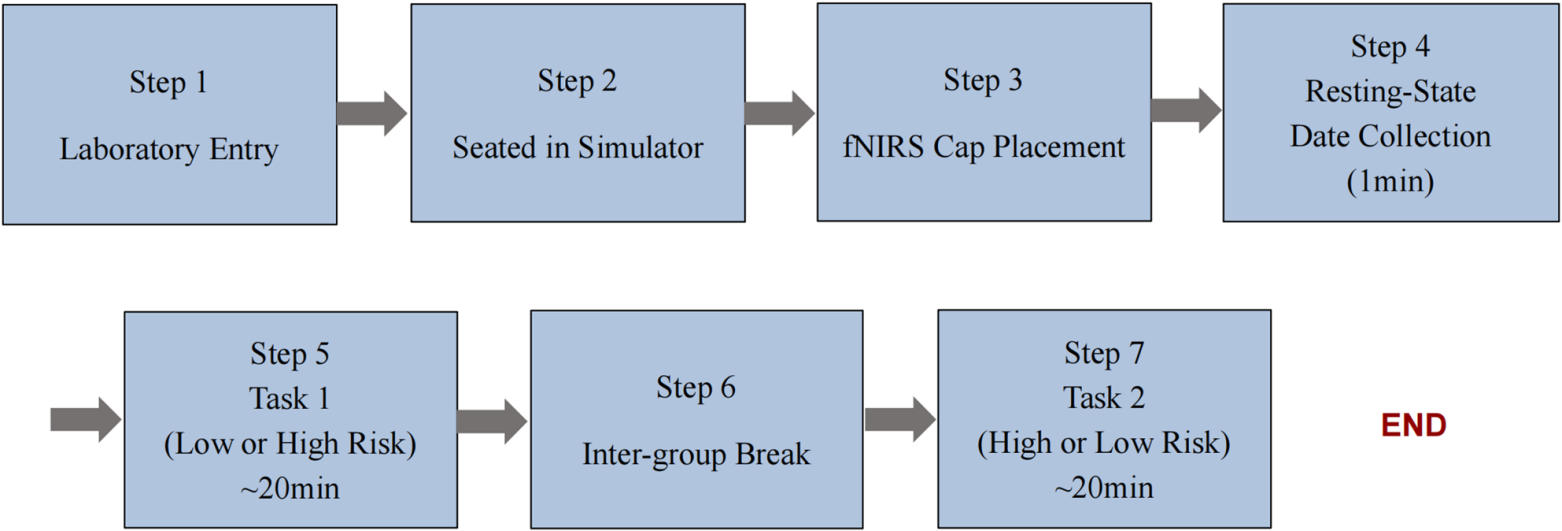
Timeline of the experimental procedure. Note. The sequence comprised (1) laboratory entry; (2) seated in the simulator; (3) fNIRS cap placement; (4) resting-state data collection (1 min); (5) task block 1 (high or low risk, ~ 20 min); (6) inter-block break (~ 5 min); and (7) task block 2 (alternative risk level, ~ 20 min). The task order was fully counterbalanced across participants (AB/BA) to prevent sequence effects

Crucially, the decision time was defined as the interval from the cessation of the risk event (end of wind shear) to the onset of the participant’s verbal report. This precise timestamping was essential for subsequent fNIRS analysis, allowing the cognitive deliberation phase to be isolated from speech-related motion artefacts. The experimenter recorded these critical event timestamps using a Bluetooth marking button. The timeline of the experimental procedure is illustrated in Fig. 4.

### Performance assessment

To provide a more comprehensive evaluation of operational decision performance, an overall performance score was derived from an expert-based behavioural assessment of flight task execution.

During the experiments, all of the simulator parameters and flight operation videos were systematically recorded and archived. These records enabled a post-experiment secondary evaluation of the participants’ operational performance.

The scoring framework was developed in consultation with two senior airline captains, each with more than 30 years of commercial aviation experience and certified flight instructor qualifications. The evaluation criteria were designed to primarily reflect decision effectiveness while capturing the operational consequences of decision execution. Specifically, the scoring incorporated three components: (a) decision quality, which indicated the appropriateness and safety of the selected operational strategy; (b) aircraft attitude control, which represented the stability and controllability of the aircraft during manoeuvre execution; and (c) landing zone precision, which described the accuracy and safety of the final landing outcome. These components were included because in aviation operations, decision quality is inherently expressed through the safety and stability of subsequent flight behaviour.

All trials were independently evaluated by two trained expert raters who reviewed the recorded flight videos after the experiment was completed. To minimise potential bias, the raters were blinded to the participants’ SPS group classification and experimental condition assignments. The raters evaluated performance using standardised scoring criteria developed by the expert panel.

Inter-rater reliability was assessed using a two-way mixed-effects intraclass correlation coefficient based on the mean ratings. The analysis indicated good inter-rater reliability (ICC = 0.745; [10]). The final overall performance score for each trial was calculated as the mean of the two raters’ scores, yielding a continuous performance index that ranged from 0 to 100.

### fNIRS data preprocessing and analysis

The fNIRS data processing strictly adhered to established guidelines and was performed using the Homer3 toolbox. Prior to the experiment, the DPF was dynamically adjusted based on each participant’s age. Raw intensity signals were then converted to optical density (OD) using the modified Beer–Lambert law, with a baseline correction applied using the mean signal of the 60-s pre-task resting state. Motion artefacts were detected using a sliding window standard deviation method (window: 5 s; threshold: 5 × MAD) and corrected using iterative principal component analysis (PCA), which retained 95% of the variance. Previous studies have validated that PCA-based spatial filtering effectively removes high-frequency spike artefacts caused by facial muscle activity, such as temporalis muscle movements associated with speech [7, 45], while residual artefacts are repaired using cubic spline interpolation. To suppress cardiac and respiratory noise, a bandpass filter (0.01–0.1 Hz) and independent component analysis (ICA) were applied [9, 36]. Haemodynamic responses were then modelled using a general linear model (GLM). To minimise speech-related motion artefacts, the regressor of the “decision phase” was defined as the time window from the cessation of the risk event to the onset of the verbal report, physically excluding jaw movements during speech. Finally, beta values (*β*) were estimated to quantify cortical activation.

### Statistical analysis

A 2 (SPS: high vs. low) × 2 (task risk level: high vs. low) mixed factorial design was employed. Mixed-design analyses of variance (ANOVAs) were conducted on the decision time, overall performance scores, and BA9 activation to examine the main and interaction effects, with Bonferroni-corrected pairwise comparisons to control for Type I error. A mediation analysis using the *PROCESS* macro (Model 4) tested the indirect effect of indecisiveness on the relationship between SPS and the decision time by employing bootstrapping with 5,000 samples and 95% confidence intervals (CIs). A moderated mediation analysis (Model 14) further examined whether BA9 activation moderated the indirect pathway, with conditional indirect effects estimated at ± 1 *SD* of BA9 activation [24, 38]. All analyses were conducted in SPSS 27.0 and MATLAB R2022b, with statistical significance set at *p* < 0.05.

## Results

### Validation of the risk-level manipulation

To assess the effectiveness of the risk-level manipulation, a paired-samples t test was conducted on the participants’ subjective stress ratings. The resul ts revealed a significant difference between the two conditions, *t*(50) = −4.78, *p* < 0.001. The participants reported significantly greater subjective stress in the high-risk condition (*M* = 3.43, *SD* = 0.67) than in the low-risk condition (*M* = 2.96, *SD* = 0.72). These findings confirm that the experimental manipulation successfully induced different levels of psychological pressure.

### Effects of SPS and task risk level on the decision time

**Fig. 5:**
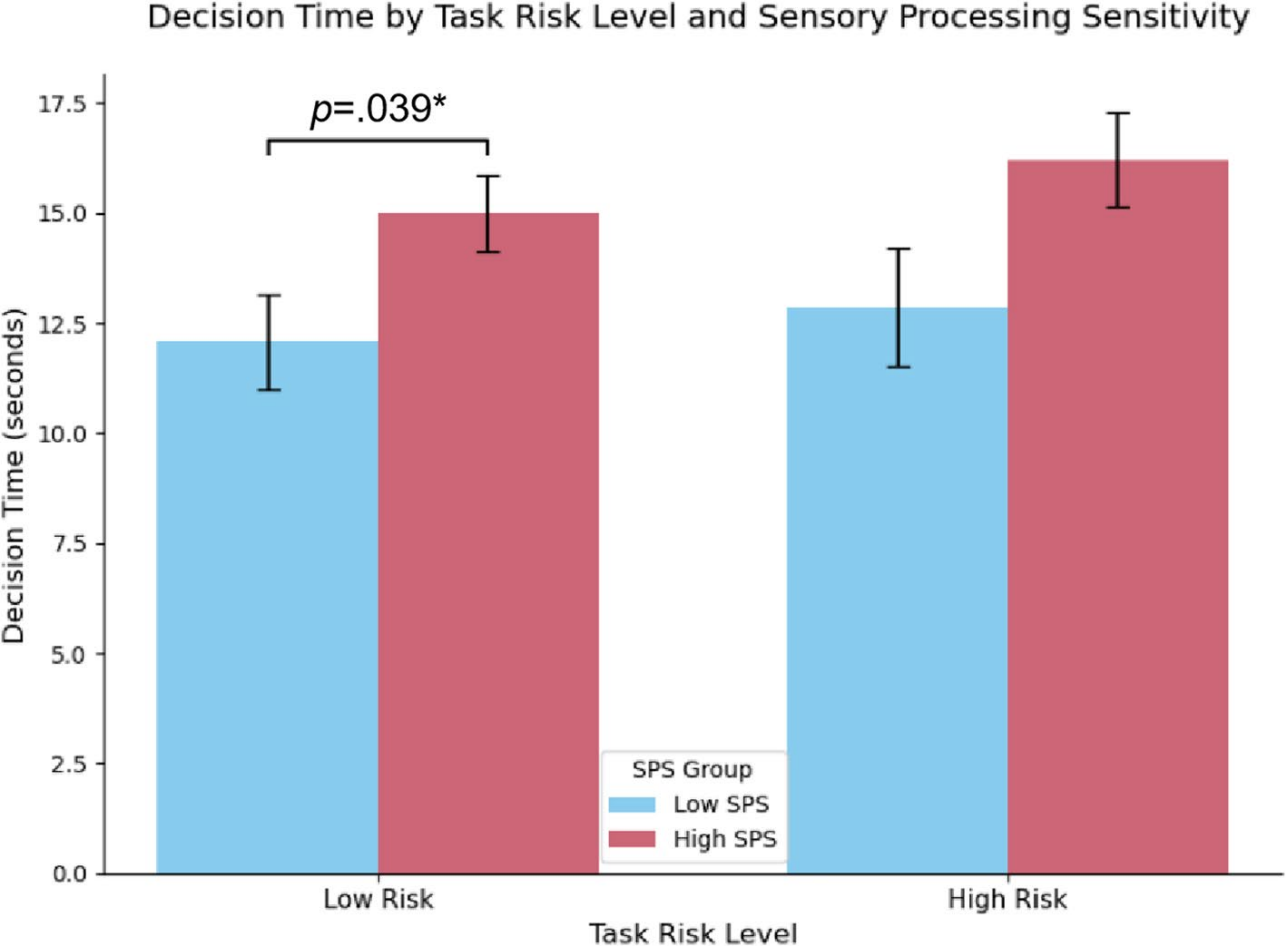
Comparison of the decision time across the SPS groups and task risk levels. Note. The bar heights represent the mean decision time (in seconds) for the high-SPS (*n* = 28) and low-SPS (*n* = 23) groups. The error bars indicate a ± 1 standard deviation (*SD*). The mixed-design ANOVA revealed a significant main effect of the SPS trait, *F*(1, 49) = 7.67, *p* =.008, *η*_*p*_.^2^ =.135, which indicated that the high-SPS group exhibited a consistently longer decision time regardless of risk. The main effect of risk (*p* =.344) and the interaction effect (*p* =.836) were not significant. * *p* <.05

A 2 (SPS: high vs. low) × 2 (task risk level: high vs. low) mixed-design ANOVA was conducted on the decision time. A significant main effect of SPS was found (*F*(1, 49) = 7.67, *p* = 0.008, *η*_*p*_^2^ = 0.135), with the participants in the high-SPS group showing longer decision times overall (see Fig. 5). No significant main effect of task risk level was found (*F*(1, 49) = 0.91, *p* = 0.344), and the interaction effect was not significant (*F*(1, 49) = 0.04, *p* = 0.836).

The descriptive statistics revealed that under the low-risk condition, low-SPS participants had a mean decision time of 12.07 s (*SD* = 5.20), whereas the high-SPS participants averaged 14.98 s (*SD* = 4.61). Under the high-risk condition, the low-SPS group averaged 12.85 s (*SD* = 6.38), and the high-SPS group averaged 16.21 s (*SD* = 5.76).

### Effects of SPS and task risk level on the overall performance score

To evaluate the decision quality and flight safety beyond mere speed, a 2 (SPS group: high vs. low) × 2 (task risk level: high vs. low) mixed-design ANOVA was conducted on the expert-rated overall performance scores. The analysis revealed significant main effects for both the SPS group, *F*(1, 49) = 11.67, *p* = 0.001, *η*_*p*_^2^ = 0.192, and task risk level, *F*(1, 49) = 134.78, *p* < 0.001, *η*_*p*_^2^ = 0.733.

**Fig. 6:**
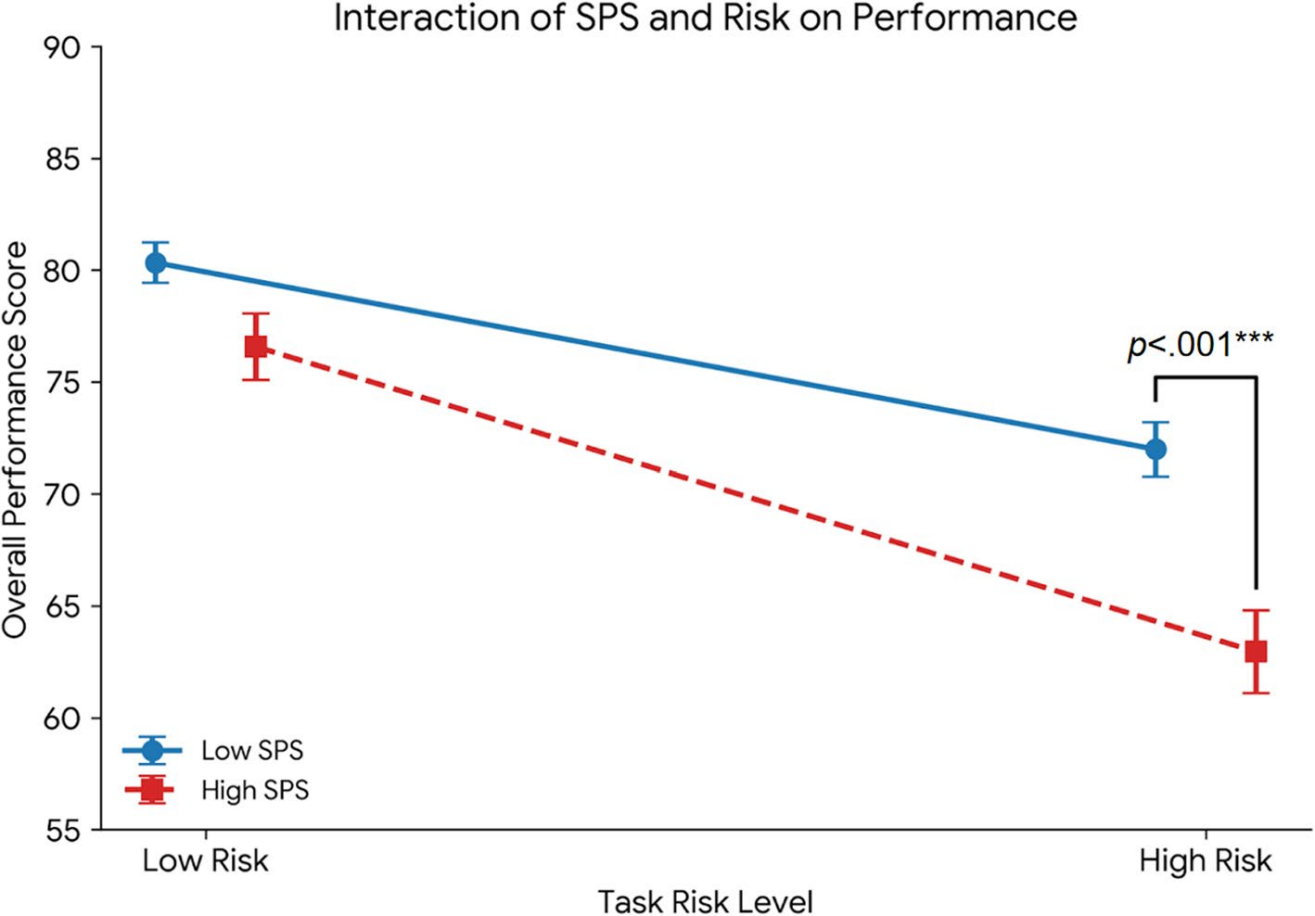
Interaction effect of SPS and task risk level on the overall flight performance scores. Note. The data points represent the mean performance scores (0–100) for the low- (n = 23) and high-SPS (n = 28) groups. The error bars indicate a ± 1 standard error of the mean (SEM). The interaction analysis (*F*(1, 49) = 7.77, *p* =.008, *η*_*p*_.^2^ =.137) revealed that although the groups performed comparably under low risk (*p* =.078), the high-SPS group exhibited a significantly greater decrease in performance under high risk than the low-SPS group did. *** *p* <.001

Crucially, a significant SPS group × task risk level interaction was observed, *F*(1, 49) = 7.77, *p* = 0.008, *η*_*p*_^2^ = 0.137. Simple effects analyses decomposed this interaction (see Fig. 6). Under the low-risk condition, the difference in performance between the high- and low-SPS groups was not statistically significant (*p* = 0.078). However, under the high-risk condition, the high-SPS group demonstrated significantly lower overall performance scores than the low-SPS group did, *t*(49) = 4.31, *p* < 0.001. Furthermore, although performance decreased for both groups from the low- to high-risk conditions, this decrease was significantly more pronounced for the high-SPS group.

### Effects of SPS and task risk level on BA9 activation

**Fig. 7:**
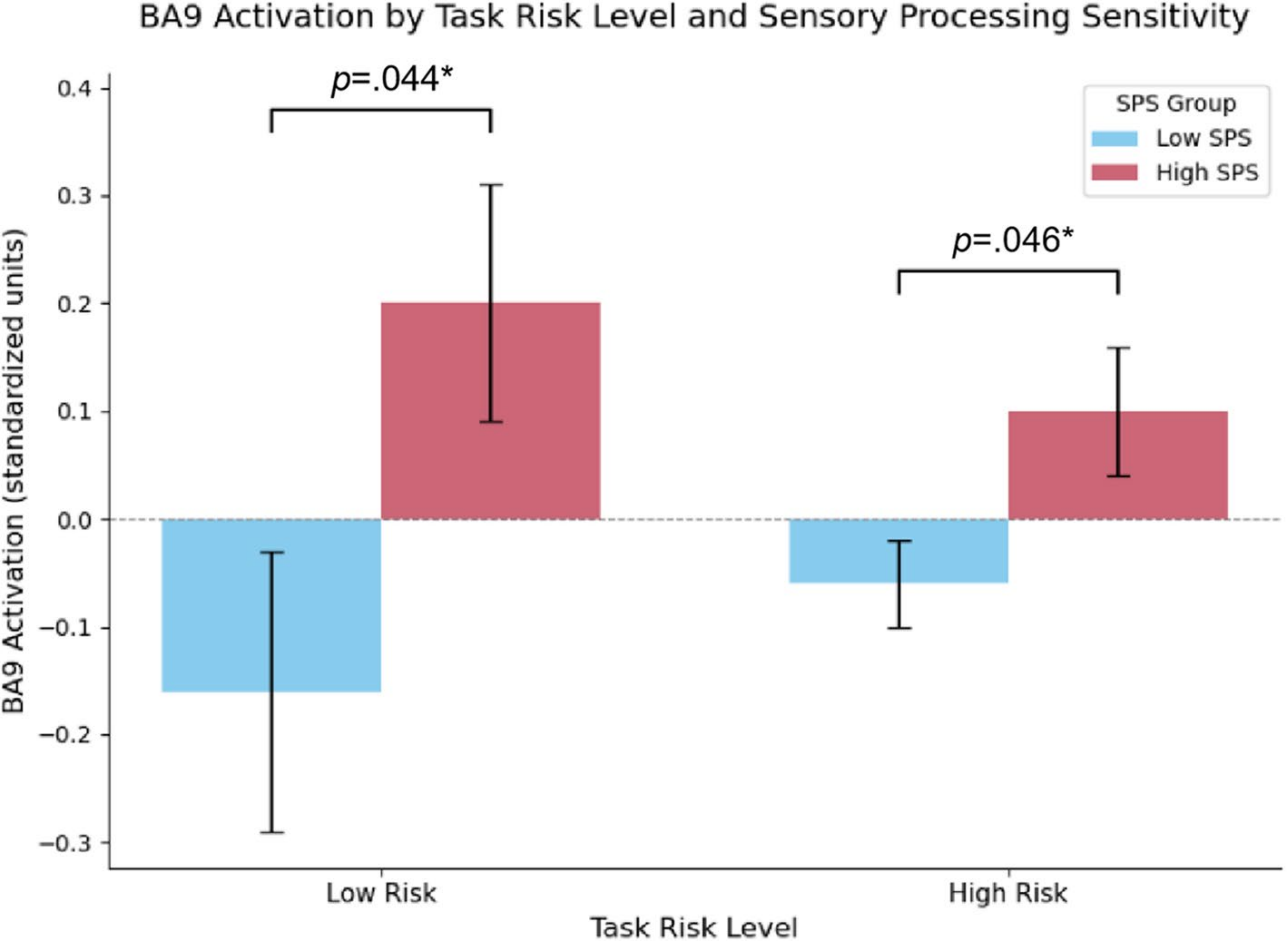
Comparison of the standardised BA9 (DLPFC) activation across SPS groups and task risk levels. Note. The error bars indicate a ± 1 standard error of the mean (SEM). A significant main effect of SPS was observed, *F*(1, 49) = 5.99, *p* =.018, *η*_*p*_.^2^ =.109, indicating that high-SPS individuals exhibited greater BA9 activation regardless of the risk condition. No significant main effect of risk (*p* =.950) or the interaction effect (*p* =.246) was found. * *p* <.05

A 2 (SPS: high vs. low) × 2 (task risk level: high vs. low) mixed-design ANOVA was conducted to examine activation in BA9 of the DLPFC. A significant main effect of SPS was found, *F*(1, 49) = 5.99, *p* = 0.018, *η*_*p*_^2^ = 0.109, indicating that the participants with higher SPS exhibited greater BA9 activation across the task conditions (see Fig. 7). However, the main effect of task risk level was not significant, *F*(1, 49) = 0.004, *p* = 0.950, and the interaction effect between SPS and task risk level was not significant, *F*(1, 49) = 1.38, *p* = 0.246.

**Fig. 8:**
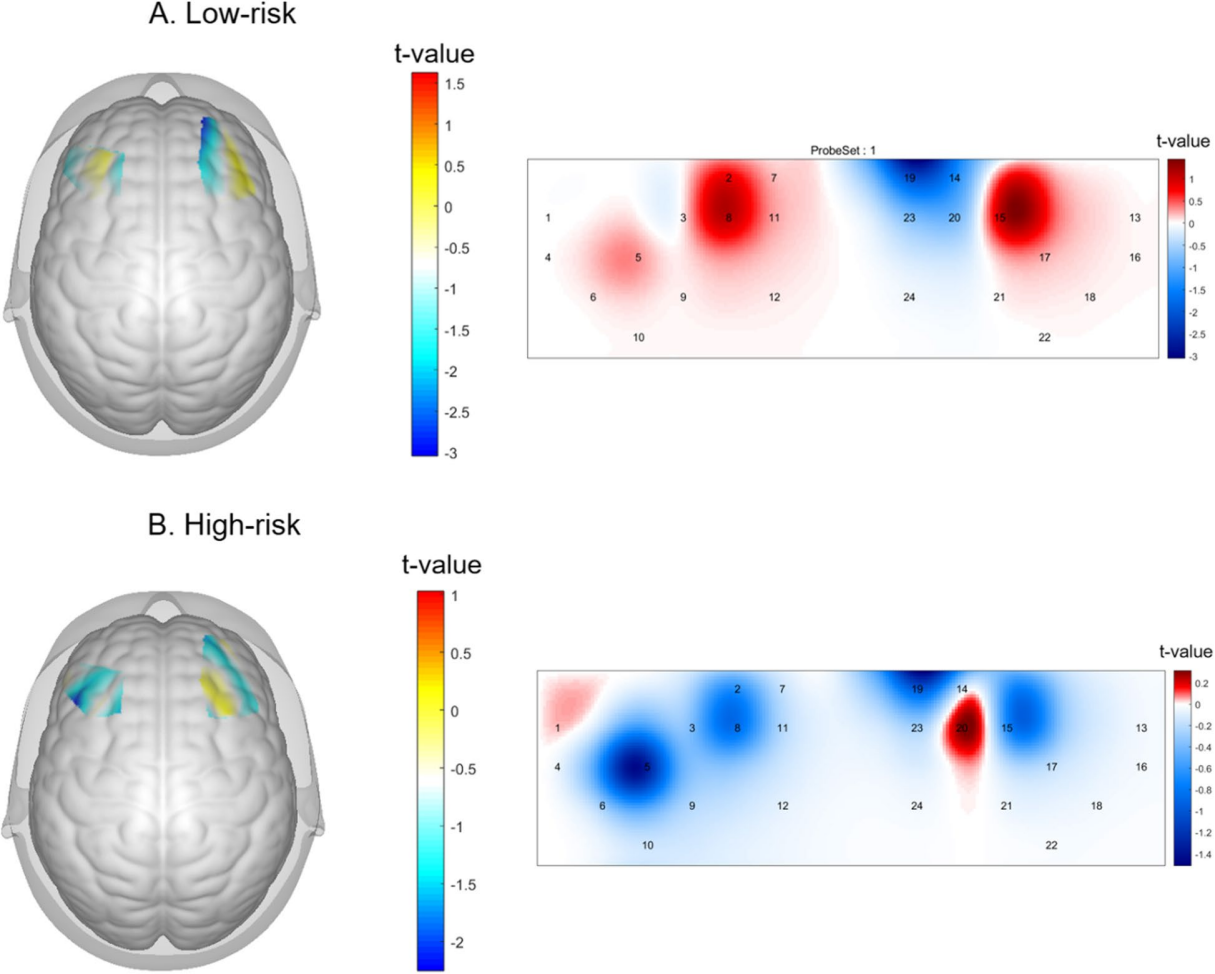
Group-level t-statistical maps of the differences in prefrontal activation between the high- and low-SPS groups. Note. The visualisation is stratified by task condition: (**A**) low-risk condition and (**B**) high-risk condition. For each panel, the left side displays the 3D surface-rendered t-map, and the right side presents the 2D interpolated t value map for the DLPFC (BA9) region of interest. The cool colours (blue/cyan) indicate the regions where the high-SPS group exhibited significantly greater activation than the low-SPS group did

The descriptive statistics revealed that under the low-risk condition, the mean standardised BA9 activation of the low-SPS group was −0.16 (*SEM* = 0.13), whereas that of the high-SPS group was 0.20 (*SEM* = 0.11). Under the high-risk condition, the mean of the low-SPS group was −0.06 (*SEM* = 0.04), and the mean of the high-SPS group was 0.10 (*SEM* = 0.06). Group-level t-statistical maps that visualise these differences in MNI space are presented in Fig. 8.

### Moderated mediation analysis

To examine the mechanisms that link SPS to the decision time, we conducted a two-step analysis. [38]. First, we tested a simple mediation model to determine whether indecisiveness mediates the relationship between SPS and the decision time. Second, we employed a moderated mediation model to investigate whether neural activation in the DLPFC (BA9) moderates the pathway from indecisiveness to the decision time. All of the continuous variables were standardised prior to analysis to mitigate multicollinearity and facilitate the interpretation of the interaction effects.

### Mediating effect of indecisiveness

The mediation analysis was performed using the SPSS PROCESS macro (Model 4) with 5,000 bootstrap resamples. The results confirmed a significant indirect effect of SPS on the decision time via indecisiveness (effect = 0.039, 95% CI [0.0031, 0.0792]). Specifically, higher SPS significantly predicted greater indecisiveness (*β* = 0.181, *p* < 0.001), which, in turn, significantly predicted a longer decision time (*β* = 0.216, *p* = 0.033). In this simple mediation model, the direct effect of SPS on the decision time was not statistically significant after controlling for the mediator (*β* = 0.051, *p* = 0.099), while the total effect remained significant (*β* = 0.090, *p* = 0.001). These findings indicate that indecisiveness mediates the impact of SPS on decision delays.

### Moderating role of BA9 activation

**Fig. 9:**
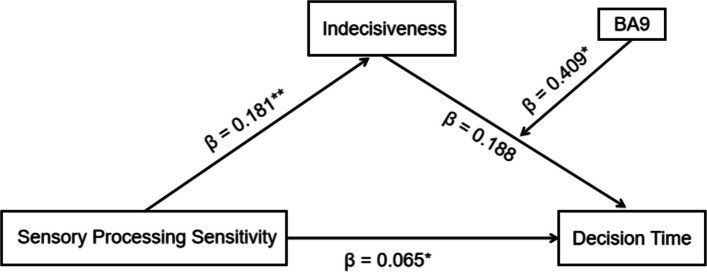
Moderated mediation model that links SPS to the decision time via indecisiveness, with BA9 activation as a moderator. Note. The values represent standardised coefficients (*β*). The interaction between indecisiveness and BA9 activation is significant (*p* =.014), which supports second-stage moderated mediation. The solid lines indicate significant paths. *p <.05. **p <.01

To test the hypothesis that BA9 activation moderates the relationship between indecisiveness and the decision time (the second stage of mediation), we utilised PROCESS Model 14. As illustrated in Fig. 9, the interaction between indecisiveness and BA9 activation was a significant predictor of the decision time (*β* = 0.409, *p* = 0.014). This finding demonstrates that the strength of the relationship between indecisiveness and the decision time varies as a function of BA9 activation.

Notably, upon the inclusion of BA9 activation and the interaction term in the model, the direct effect of SPS on the decision time became statistically significant (*β* = 0.065, *p* = 0.043).

Further analysis of the conditional indirect effects (see Table 3) revealed that the indirect pathway (SPS → indecisiveness → decision time) was not significant at low levels of BA9 activation (−1 *SD*). However, the indirect effect became significant at the mean and high levels (+ 1 *SD*) of BA9 activation. These results support a moderated mediation model and imply that the tendency for indecisiveness to prolong the decision time is amplified in individuals with elevated BA9 activation.

**Table 3:** Conditional Indirect Effects of SPS on the Decision Time at Different Levels of BA9 Activation

Mediator	Level of Moderator (BA9)	Value	Effect	Boot SE	Boot LLCI	Boot ULCI
Indecisiveness	Low (− 1 *SD*)	− 0.457	0.000	0.025	− 0.046	0.052
	Mean	0.033	0.037	0.020	0.000	0.076
	High (+ 1 *SD*)	0.523	0.073	0.024	0.023	0.119

*Boot SE* bootstrap standard error, *Boot LLCI and ULCI* lower and upper bounds, respectively, of the 95% bootstrap confidence interval. The bootstrap method used was percentile bootstrapping with 5,000 resamples

## Discussion

### Interpretation of the findings

In this study, flight simulation, behavioural measures, and fNIRS were combined to examine how SPS influences pilots’ decision-making under stress. We tested the mediation pathway of *SPS → indecisiveness → decision time* and the moderating role of the DLPFC (BA9) activation. The results show that individual differences in SPS can directly affect flight decision performance, with potential implications for aviation safety.

Consistent with Hypothesis 1, the high-SPS pilots exhibited significantly longer decision times during simulated emergencies. This conclusion suggests that heightened sensitivity to external cues slows decision-making during critical flight phases [1, 4]. In practice, such delays may lead to hesitation during high-risk manoeuvres—such as go-arounds in adverse weather—where timely action is crucial for accident prevention [16]. High-SPS individuals are more susceptible to cognitive overload from multiple cockpit inputs (e.g., warnings, instruments, and ATC communication), which impedes information integration and decision execution [30, 33, 39]. These findings align with previous human factor research that reveals that higher sensitivity increases vulnerability to distraction and reduces decision efficiency [28, 29, 33]. Unlike prior studies that focus primarily on stress or workload, the present study integrates personality traits, behavioural tendencies, and neural responses, highlighting SPS as a measurable trait with direct relevance for aviation safety management.

The results also support Hypothesis 2 and demonstrate that indecisiveness mediated the effect of SPS on the decision time. It is crucial to distinguish conceptually between the trait and the outcome: SPS functions as an input-oriented “information processing style” characterised by deep cognitive reflection [4], whereas indecisiveness represents the specific “behavioural output” that occurs when this processing style depletes cognitive resources under time pressure [17]. Consequently, SPS slows decision-making indirectly through a decision style characterised by hesitation and difficulty committing under stress [15, 17]. Operationally, highly sensitive pilots may delay actions during emergencies not because of technical incompetence but because of a risk-avoidant tendency driven by obligatory deep processing. Although this deep processing is intended to prevent errors, such hesitation effectively reduces the temporal window for safe manoeuvres. In aviation contexts where seconds are critical, indecisiveness acts as the behavioural pathway through which SPS manifests as delayed action. This mechanism is comparable to findings in fatigue research, where protective behaviours mediate the link between fatigue and safety outcomes [12].

Hypothesis 3 is thus supported. The results of the moderated mediation analysis signify that BA9 activation strengthened the association between indecisiveness and the decision time and suggest that BA9 plays a regulatory role in determining how internal decisional conflict translates into behavioural delay.

Within the present task context, high-SPS pilots are more likely to experience intensified conflict between internally oriented cognitive processing (e.g., reflection, self-monitoring, and situational evaluation) and the operational requirements for rapid external action. As a key subregion of the DLPFC, BA9 has been implicated in integrating internally generated cognitive activity with externally directed executive control systems [25, 31], positioning it as a neural substrate for resolving decisional conflict.

From a mechanistic perspective, increased BA9 activation is interpreted as reflecting compensatory regulatory effort recruited to manage heightened internal conflict. Sustained engagement of such regulatory processes is likely to impose increased neural processing costs, which may manifest as reduced network efficiency under high-demand conditions; this is consistent with the neural efficiency hypothesis that implies that increased neural recruitment without corresponding performance gains reflects reduced processing efficiency [14, 32]. Within this process–outcome framework, elevated regulatory effort represents the dynamic control process, whereas reduced neural efficiency identifies its functional consequences.

Accordingly, the present findings suggest a sequential mechanism in which heightened compensatory regulatory effort increases the neural processing cost, thereby amplifying the translation of indecisiveness into observable decision delay. Under a lower regulatory demand, hesitation may be resolved without substantial behavioural cost; however, when regulatory load increases, indecisiveness is more likely to manifest as a prolonged decision time.

### The dissociation between subjective stress and the decision time

The dissociation between the subjective stress ratings, which significantly increased under the high-risk conditions, and the objective decision time warrants a specific discussion. We argue that the null main effect of risk on the decision time does not imply a ceiling effect or a failure of experimental manipulation. In fact, the overall flight performance score revealed a significant main effect of risk and a risk × SPS interaction, which confirms that the task difficulty was well calibrated and sensitive to operator ability.

With respect to the null effects on the decision time, the descriptive statistics show that the high-SPS individuals were consistently slower than the low-SPS individuals across both the low-risk (*M*_*High*_ = 14.98 s vs. *M*_*Low*_ = 12.07 s) and high-risk (*M*_*High*_ = 16.21 s vs. *M*_*Low*_ = 12.85 s) conditions. This consistent latency gap (~ 3 s) suggests that the “cautious processing style” is a stable trait of high-SPS individuals that persists regardless of the situational risk [40], which may account for the insignificant interaction between risk and SPS (*p* = 0.83). Furthermore, the lack of a main effect of risk on the decision time suggests a strategic trade-off: the participants likely prioritised maintaining their decision speed to meet emergency demands (adhering to standard operating procedures), whereas the cognitive cost of the high-risk scenario was transferred to the execution quality, as evidenced by the significant decline in flight performance.

### Integrated impact on the decision speed and overall performance

The present study reveals a dual burden associated with high SPS in aviation emergencies characterised by prolonged decision-making and reduced overall flight performance. This pattern may reflect a compensatory resource allocation process under an elevated operational workload. In real-world aviation contexts, pilots are trained to prioritise timely decision-making in accordance with standard operating procedures (SOPs), as delayed responses during emergencies may substantially increase safety risk. Under high-risk conditions, maintaining decision timeliness may therefore draw cognitive resources away from fine motor control, situational monitoring, and execution precision, resulting in reduced overall flight performance.

These findings further suggest that high-SPS individuals may be particularly vulnerable to this compensatory imbalance. Although they appear capable of maintaining adequate performance under a moderate workload, compounded emergency stressors may exceed their regulatory capacity and lead to inefficient cognitive resource allocation and reduced operational effectiveness.

### Practical implications

From a practical perspective, the present findings provide new insights into pilot training and performance management by identifying a neurocognitive pathway through which SPS influences the decision time via indecisiveness. These findings offer a trait-based perspective for understanding individual variability in pilot decision-making performance.

Importantly, the results support a shift from personnel selection towards individualised training support. Because SPS represents a stable personality trait rather than a pathological vulnerability, performance differences observed among high-SPS pilots are more appropriately addressed through targeted cognitive regulation training.

Mindfulness-based interventions (MBIs) represent a promising approach, as prior research has demonstrated that mindfulness training can enhance executive control and improve the efficiency of prefrontal regulatory networks [3, 35, 41]. From an applied perspective, brief mindfulness-based attentional regulation exercises could be incorporated into existing crew resource management (CRM) and emergency management and control (EMC) training. For example, short pre-simulator attentional stabilisation exercises and guided attentional control strategies during emergency simulation scenarios may help pilots maintain cognitive flexibility under high workloads.

For high-SPS pilots, such training may support BA9-related regulatory efficiency and reduce indecisiveness during time-critical flight operations. More broadly, integrating psychological regulation strategies into pilot training programmes may complement traditional technical skill development by supporting trait-sensitive cognitive performance optimisation.

## Limitations and conclusion

Several limitations of this study should be acknowledged. First, the sample consisted of cadet pilots whose decision-making processes may differ from those of experienced commercial pilots; future research should include a wider range of flight experience to test generalizability. Second, although simulators provide controlled settings for studying emergencies, they cannot fully reproduce the physiological stress of real flight. Third, this study focused on prefrontal activity, whereas real-time decision-making likely involves broader neural networks related to emotion and conflict monitoring.

A key limitation of the present study concerns the relatively modest sample size (N = 51). Although an a priori power analysis for complex moderated mediation models suggested that a larger sample (approximately N = 150) would be desirable [24], the final sample size reflects a deliberate recruitment strategy that prioritised population homogeneity and methodological control.

The participants were drawn from a finite and operationally constrained cohort of flight cadets (approximately N = 200) at the Civil Aviation University of China who were transitioning into the flight training phase. To maximise the study’s internal validity and experimental standardisation, recruitment was restricted to cadets within a single training cohort. Expanding recruitment across different academies or training stages may have increased the sample size but would likely have introduced substantial confounding variability, including differences in training procedures, simulator fidelity, instructional environments, and fatigue exposure.

Importantly, sample sizes of a similar magnitude are commonly reported in ecological neuroergonomics and aviation fNIRS research. For example, prior high-fidelity flight simulation studies have employed samples ranging from 18 to 28 pilots [19, 42–44]. Nevertheless, the present sample remains smaller than that recommended for detecting subtle interaction effects in complex statistical models, which may increase the risk of Type II errors and potential model overfitting. A post hoc sensitivity analysis using G*Power 3.1 indicated approximately 80% power to detect medium-to-large effects (*f*^2^ = 0.2657), while the power for smaller effects was limited.

Accordingly, the findings, particularly the absence of certain behavioural interaction effects, should be interpreted cautiously. Future research should seek to replicate and extend the present findings using larger, multi-site pilot cohorts to increase statistical power and generalizability.

Accordingly, the results of the present study provide preliminary evidence that SPS is associated with a prolonged decision time during simulated emergency flight tasks. The findings suggest that indecisiveness mediates the relationship between SPS and decision latency, whereas DLPFC (BA9) activation moderates the latter stage of this pathway.

Conducted within a high ecological-validity flight simulation environment, this study contributes to understanding the neurocognitive mechanisms through which individual trait differences influence aviation decision-making performance. These findings highlight the potential importance of incorporating trait-sensitive and psychologically informed approaches into pilot training. In particular, interventions that target cognitive regulation, such as mindfulness-based training, may represent a promising avenue for supporting decision performance in high-demand flight scenarios. Nevertheless, given the specialised sample and modest sample size, future research that employs larger and multi-site pilot cohorts is needed to replicate and extend these findings.

## Data Availability

The data that support the findings of this study are available from the corresponding author upon reasonable request.

## Abbreviations

ACT: Attentional Control Theory
AGL: Above Ground Level
BA9: Brodmann Area 9
DLPFC: Dorsolateral Prefrontal Cortex
DT: Decision Time
EEG: Electroencephalography
EFA: Exploratory Factor Analysis
EICAS: Engine Indication and Crew Alerting System
FFS: Full Flight Simulator
fMRI: Functional Magnetic Resonance Imaging
fNIRS: Functional Near-Infrared Spectroscopy
GLM: General Linear Model
HbO: Oxygenated Hemoglobin
HSPS: Highly Sensitive Person Scale
IAF: Initial Approach Fix
ICA: Independent Component Analysis
ICC: Intraclass Correlation Coefficient
IS: Indecisiveness Scale
MBI: Mindfulness-Based Interventions
MNI: Montreal Neurological Institute
NEH: Neural Efficiency Hypothesis
PCA: Principal Component Analysis
SPS: Sensory Processing Sensitivity
